# Correspondence: systematic reviews do not always capture context of real-world intervention programmes for childhood obesity (response to Littlewood, et al., 2020 in BMC Public Health)

**DOI:** 10.1186/s12889-021-10486-5

**Published:** 2021-03-15

**Authors:** Cervantée E. K. Wild, Tami L. Cave, Esther J. Willing, José G. B. Derraik, Cameron C. Grant, Paul L. Hofman, Yvonne C. Anderson

**Affiliations:** 1 Department of Paediatrics: Child and Youth Health, Faculty of Medical and Health Sciences, University of Auckland, Auckland, New Zealand; 2 Tamariki Pakari Child Health and Wellbeing Trust, New Plymouth, New Zealand; 3 Liggins Institute, University of Auckland, Auckland, New Zealand; 4 Kōhatu – Centre for Hauora Māori, University of Otago, Dunedin, New Zealand; 5 Starship Children’s Hospital, Auckland District Health Board, Auckland, New Zealand; 6 Department of Paediatrics, Taranaki District Health Board, New Plymouth, New Zealand

**Keywords:** Obesity, Systematic review, Intervention, Child, Adolescent, Oceanic ancestry group

## Abstract

In a recent issue of the BMC Public Health journal, Littlewood et al. described the results of a systematic review of interventions to prevent or treat childhood obesity in Māori or Pacific Island peoples. They found that studies to date have had limited impact on improving health outcomes for Māori and Pacific Island peoples, and suggest this may be due to a lack of co-design principles in the conception of the various studies. Ensuring that interventions are appropriate for groups most affected by obesity is critical; however, some inaccuracies should be noted in the explanation of these findings. There is a risk with systematic reviews that the context of intervention trials is lost without acknowledging the associated body of literature for programmes that refer to the ongoing commitment to communities and groups most affected by obesity.

## Main text

Systematic reviews face considerable challenges when considering context within the evidence synthesis process. Many complex interventions, such as those interventions to prevent or treat childhood obesity, rely on a wider body of literature to evaluate their impact than reports of randomised controlled trials and study protocols [1].

In issue 20 of BMC Public Health, Littlewood et al. described the results of a systematic review of interventions to prevent or treat childhood obesity in Māori or Pacific Island peoples, who experience disproportionately higher rates of childhood obesity than their non-Māori, non-Pacific Island counterparts [2]. Overall, the authors found that the included studies had limited impact with respect to improving anthropometric and secondary outcomes, including “cardio-metabolic” and psychological outcomes [2]. We agree that addressing obesity in population groups most affected by childhood obesity is critical. However, evaluation of these interventions requires taking into account the associated literature providing the circumstances in which the intervention was embedded (i.e. context); both qualitative and mixed-methods research contribute towards understanding the effectiveness of interventions by providing wider context [1]. In this letter, we would like to clarify some inaccuracies in the description of the Whānau Pakari programme and respond to several assertions made by authors pertaining to the programme in the review.

The authors stated that physical activity was the primary focus of the Whānau Pakari programme, thereby assuming the null anthropometric outcome was the result of a lack of dietary intervention intensity [2]. However, the Whānau Pakari trial assessed a multi-disciplinary programme focused holistically on achieving persistent healthy lifestyle change. A wide range of health and wellbeing outcome measures were included at each assessment, including anthropometric, dietary, physical activity, and psychological outcome measures [3–5]. The programme involved weekly sessions at community venues, in order to ‘demedicalise’ the programme, taking it outside of hospital walls and into the community. These sessions included dietary sessions (such as recipe makeovers, virtual supermarket shopping, planting vegetable gardens, food label reading, and cooking sessions), family physical activity sessions, and psychology sessions across a 12-month period [6, 7].

The assertion that consultation with Māori stakeholders was limited to the project conception is also incorrect. Consultation with the community and with Māori stakeholders continued throughout the trial [7], and is ongoing in the Whānau Pakari service and research programme.

The Whānau Pakari programme was developed after an audit demonstrated that the previous programme in the region was not meeting the needs of Māori in terms of access and outcomes [8]. The authors of this systematic review surmise that the higher dropout rate for Māori in Whānau Pakari was due to a lack of robust co-design methodologies in the conception of the trial (and therefore presumably a lack of specificity and tailoring to Māori and Pacific Island participants) [2]. Indeed, at the time of publication of the 12-month results, we suggested that greater incorporation of a Māori worldview, as well as reviewing the intervention model and location, could potentially improve engagement for Māori [6]. However, research into the barriers and facilitators of engagement in Whānau Pakari has determined that this is not necessarily the case [9]. A survey of past Whānau Pakari participants (45% of whom were Māori) identified that competing priorities and socioeconomic challenges were key reasons for low programme attendance. Further, while the programme was considered culturally appropriate by almost all participants, Māori more frequently reported that past experiences of health care influenced their willingness to engage [9].

Achieving equitable outcomes for Māori and Pacific Island peoples is of critical importance; therefore, there is ongoing evaluation of the Whānau Pakari programme in this regard. Our most recent research can perhaps provide some more context to this review. A study of 64 in-depth interviews with equal numbers of Māori and non-Māori families has since shown that inequities in programme engagement for Māori were due to substantial upstream socioeconomic barriers, and a distrust of health services due to historical experiences of care elsewhere in the system. By contrast, Whānau Pakari was considered to be culturally appropriate by participants. Respectful and appropriate care with an emphasis on positive relationship-building may be a way to partially mitigate these upstream effects, although it was acknowledged that this was not always sufficient to retain families who were dealing with multiple complex challenges [10, 11].

We propose there is a risk with systematic reviews that the context of trials is lost without acknowledging the associated body of literature for programmes that refer to the ongoing commitment to communities and groups most affected by obesity, especially when mixed-methods research is being undertaken. The Preferred Reporting Items for Systematic reviews and Meta-analyses (PRISMA) reporting standards reference ‘context’ in terms of the circumstances requiring the review itself, rather than referencing the contexts of studies included in the review [12]. The PRISMA extension for Complex Interventions includes the elements of ‘time’ and ‘setting’ [13], but this still does not account for the supporting information found in the wider body of associated literature that is presently not captured in systematic reviews of intervention trials. Alternative systematic review methods, such as realist synthesis or meta-ethnography, may allow for exploration of context throughout the review [1] and may allow for the inclusion of supporting literature.

Finally, we agree that co-designed and community-partnered interventions are likely to produce more positive outcomes for those who are most affected by obesity. A multipronged approach to address health system access barriers upstream is also required to reduce inequities in attendance and outcome for Māori and Pacific Island peoples. Interventions such as those described in Littlewood et al.’s systematic review may be culturally tailored, appropriate, co-designed, and culturally safe; yet, if upstream determinants of health and wider socioeconomic disparities are not addressed, it is likely that inequities in outcome and engagement for Māori and Pacific Island peoples will continue to persist [10, 11].

## Data Availability

Not applicable.

## Abbreviation

PRISMA: Preferred Reporting Items for Systematic reviews and Meta-analyses
